# Serum Carnosinase 1 Is Not Associated with Insulin Resistance or Glucose Metabolism in a Type 1 Diabetes Cohort

**DOI:** 10.3390/biomedicines13020366

**Published:** 2025-02-05

**Authors:** Jiedong Qiu, Benito A. Yard, Bernhard K. Krämer, Harry van Goor, Peter R. van Dijk, Aimo Kannt

**Affiliations:** 1 5th Medical Department, University Hospital Mannheim, Heidelberg University, 68167 Mannheim, Germany; jiedong.qiu@medma.uni-heidelberg.de (J.Q.); benito.yard@medma.uni-heidelberg.de (B.A.Y.); bernhard.kraemer@umm.de (B.K.K.); 2 Department of Pathology and Medical Biology, University Medical Centre Groningen, University of Groningen, 9713 GZ Groningen, The Netherlands; h.van.goor@umcg.nl; 3 Department of Endocrinology, University Medical Centre Groningen, University of Groningen, 9713 GZ Groningen, The Netherlands; 4 Isala Diabetes Centre, 8025 AB Zwolle, The Netherlands; 5 Fraunhofer Institute for Translational Medicine and Pharmacology ITMP, 60596 Frankfurt, Germany; 6 Institute for Clinical Pharmacology, Goethe University Frankfurt, 60596 Frankfurt, Germany

**Keywords:** antioxidants, carnosinase 1, diabetic nephropathy, observational study, type 1 diabetes mellitus

## Abstract

**Background/Objectives**: Preclinical studies suggest that the deleterious effect of a high serum carnosinase 1 (CN1) concentration is attributed to its adverse effects on insulin sensitivity and glucose metabolism. However, there is little evidence for a modulating role of CN1 in glucose metabolism in humans. **Methods**: We measured serum CN1 concentration in an observational type 1 diabetes cohort of 172 patients in whom glucose variability (MAGE, MODD, SD of individual blood glucose, mean, and CV) was recorded by blinded continuous glucose monitoring for 5–7 days. Furthermore, insulin dose per kg body weight was compared. **Results**: Insulin sensitivity (insulin dosage) and glucose variability parameters did not differ between different CN1 tertiles (*p* > 0.05). **Conclusions**: There was no association of serum CN1 with indices of glucose variability in this type 1 diabetes cohort.

## 1. Introduction

Diabetic kidney disease (DKD), defined as chronic kidney disease in a person with diabetes, is one of most severe complications of diabetes and is associated with substantial loss of quality of life and reduced life expectancy. It is estimated that approximately 20–50% of people with type 2 diabetes (T2D) will develop DKD; this value is 30% for people with type 1 diabetes (T1D) [1,2]. Worldwide, DKD is the leading cause of chronic kidney disease and end-stage kidney disease, accounting for 50% of cases [1,2]. A better understanding of factors leading to the development of DKD may help improve prevention and therapy.

The serum enzyme carnosinase 1 (CN1) has been suggested to be involved in the pathogenesis of DKD [3,4,5,6]. CN1 is a serum dipeptidase, which degrades carnosine, homocarnosine, and anserine. These dipeptides protect against formation of advanced glycation (AGE) and lipoxidation end products (ALE) [3] and were shown to ameliorate hyperglycemia, insulin resistance, and albuminuria in rodent models [4,5,6]. Increased CN1 expression is also associated with worse glycemic control and renal impairment [4,7,8,9].

Although the association between CN1 and DKD has been demonstrated in several studies, its mechanism of action is still unclear. It has been suggested that CN1 is linked to altered insulin sensitivity and glucose metabolism. Indeed, in animal models, the effect of CN1 overexpression on DKD was associated with impaired glucose control [7,10], even in models that showed little or no effect on DKD [4,11]. Supplementation of its major substrate, carnosine, can not only improve glucose metabolism in animal models of obesity, diabetes and DKD [4,6,12,13], but also in human trials, where carnosine reduced HbA1c and fasting glucose and prevented worsening of insulin resistance [14,15,16,17]. 

Based on previous studies, we hypothesized that glucose variability may be related to serum CN1 concentration, and, therefore, we measured serum CN1 concentrations in a type 1 diabetes cohort, in which glucose variability was assessed using a continuous glucose monitor for 5–7 days.

## 2. Materials and Methods

### 2.1. Cohort

This was an observational case–control study, which took place at the Isala hospital (Zwolle, The Netherlands) and the Diaconessenhuis hospital (Meppel, The Netherlands) between June 2012 and March 2014. The initial aim of the study was to compare different insulin application routes in regard to glycemic control and variability [18]. The primary outcome of the study was the difference in HbA1c levels between groups, assessed in a non-inferiority analysis. The selection of the patients was previously described in detail [18]. In short, patients with T1D with either an implanted insulin pump for continuous intraperitoneal insulin infusion (CIPII) or subcutaneous (SC) insulin treatment for the past 4 years without interruptions of >30 days were included. Patients were also included if they were between 18 and 70 years of age, had an HbA1c level of >7.5% (58 mmol/mol; for peritoneal insulin pump)/7.0% (53 mmol/mol; for subcutaneous insulin treatment) and/or had five or more hypoglycemic events (<4 mmol/L) per week. CIPII is a treatment option for selected patients with type 1 diabetes mellitus that fail to achieve adequate glycaemic control or have frequent hypoglycaemic episodes with subcutaneous insulin administration (by multiple daily injections or continuous subcutaneous insulin infusion with an insulin pump).

Patients with impaired renal function (plasma creatinine ≥ 150 µmol/L or glomerular filtration rate as estimated by the Cockcroft–Gault formula ≤ 50 mL/min) were excluded, since renal impairment might influence glucose variability. In addition, people were excluded in case of cardiac problems (unstable angina or myocardial infarction within the previous 12 months or NYHA class III or IV congestive heart failure), cognitive impairment, current or past psychiatric treatment for schizophrenia, cognitive or bipolar disorder, current use of oral corticosteroids, if they were suffering from a condition, which necessitated corticosteroid use more than once in the previous 12 months, alcohol or drug abuse, and patients with current gravidity or plans to become pregnant during the study.

A blinded continuous glucose monitor (CGM; iPro^®^ from Medtronic, Dublin, Ireland) was attached during the first initial clinical visit and removed 5–7 days later. During the second clinical visit, clinical parameters were measured, and blood sampling was performed. From the initial 183 patients, sufficient serum could be obtained from 172 patients.

### 2.2. CN1 Concentration Measurement

We measured serum CN1 in the patient samples acquired during their second clinical visit in a total of 172 patients using the commercially available ELISA, CSB-EL005639HU from Cusabio Technology (Houston, TX, USA), following the manufacturer’s instructions. 

### 2.3. Renal Function and Serum Insulin

The following parameters were used for the current study: age, gender, body mass index (BMI), diabetes duration, glycated hemoglobin (HbA1c, expressed in mmol/mol as advised by the International Federation of Clinical Chemistry), systolic blood pressure (SBP), diastolic blood pressure (DBP), plasma creatinine, estimated glomerular filtration rate (eGFR CKD-EPI), urinary albumin creatinine ratio (ACR), and the insulin demand per kg bodyweight. The clinical characteristics of the cohort are shown in Table 1.

### 2.4. Glucose Variability

All patients were requested to perform four blood glucose self-measurements daily during the CGM period using Contour® XT (Bayer Healthcare AG, Leverkusen, Germany) to calibrate the CGM device. Interstitial glucose profiles were monitored using a blinded CGM. The CGM was placed in the periumbilical area and for pump users contralateral to the pump (CIPII). Patients that used insulin injections were instructed not to inject insulin on the same side where the sensor was attached. 

Since continuous intraperitoneal insulin infusion (CIPII) was used as a last-resort treatment, patients with CIPII were, thus, expected to have higher mean glucose levels. The coefficient of variation (CV), standing for intraday variation in glucose levels and defined as the standard deviation (SD) of the individual glucose per day divided by the mean of glucose values, was chosen as the primary parameter for glucose variability. Additional parameters of glucose variability were extracted and analyzed; the mean amplitude of glucose excursions (MAGE) was defined as the mean absolute difference between glycemic oscillation (difference between peak and nadir exceeding 1 SD). Furthermore, as a measure of interday variation, the mean of daily differences (MODD), defined as the mean absolute difference between glucose values taken on two consecutive days at the same timepoints, was analyzed. CGM data were processed using the GV calculation tool (GlyVarT; Medtronic, Dublin, Ireland). For comparison, the average and SD of blood glucose are also displayed in Table 1.

### 2.5. Statistics

SPSS Statistics V26 for Windows (IBM, Armonk, NY, USA) was used for all statistical analyses. A two-sided alpha level of less than 0.05 was considered significant. Conducting a sample size calculation was challenging because the expected effect size (i.e., the influence of CN1 on insulin sensitivity and glucose metabolism in T1D patients) was unknown. For the correlation analysis and cohort size in our study, a correlation coefficient of r = 0.22 (or r^2^ = 0.048) would have been detectable with a two-tailed alpha of 0.05 and a beta of 0.20.All variables were checked for normality with normality plots before analysis. Outliers were detected on a boxplot but only eliminated in case of a multivariable outlier. Since serum CN1 seemed to be non-normally distributed, we divided it into three tertiles (*n* = 57/58 each group) and compared renal function, serum insulin, and glucose variability between the tertiles using one-way ANOVA if normality for each group could be assumed, otherwise the non-parametric Kruskal–Wallis test was used. Gender proportions were compared using the chi-squared test. Continuous parameters were shown as mean (SD) or as median [IQR for interquartile range]. Nominal data were shown as absolute number (percentage). Missing data were excluded pairwise from the analyses. 

### 2.6. Ethics Approval

The study protocol was approved by the local medical ethics committee, and all patients gave informed consent (identifier: 12.0766, date of approval 6 November 2012). Furthermore, the study protocol was also registered prior to the study at ClinicalTrials.gov (NCT01621308) and at the ToetsingOnline.nl from the Dutch Central Committee on Research Involving Human Subjects (NL41037.075.12). 

## 3. Results

### 3.1. Patient Characteristics

Clinical characteristics of study participants are summarized in Table 1. The mean age within this cohort of T1D patients was 50.3 ± 12.5 years, with a mean duration of T1D of 26.3 ± 12.3 years. In total, 63% of study participants were male. Mean HbA1c was 63.7 ± 10.2 mmol/mol, corresponding to 8 ± 3%. Patients did not have signs of diabetic kidney disease: plasma creatinine, estimated glomerular filtration rate, and urinary albumin-to-creatinine ratio were in the normal range.

### 3.2. Insulin Application Routes

Carnosinase 1 (CN1) concentration was measured in 172 patients with T1D. Since normality cannot be assumed based on normality plots and the Shapiro–Wilk test (*p* < 0.001), the non-parametric Kruskal–Wallis test was performed to assess serum CN1 concentration in patients with SC insulin application and CIPII (*n* = 135 and 37, respectively), and no significant difference was detected (*p* = 0.24).

### 3.3. Serum CN1 Concentration and Patients’ Characteristics 

As presented in Table 1, there was no significant difference in patient characteristics between serum CN1 tertiles (*p* > 0.05). In regard to renal function parameters, neither eGFR CKD-EPI nor microalbuminuria significantly differed between CN1 tertiles (*p* > 0.05). 

### 3.4. Insulin Sensitivity and Glucose Control

The insulin demand per kg body weight did not differ between serum CN1 tertiles (*p* > 0.05) and there was also no difference in mean blood glucose, standard deviation of blood glucose, coefficient of variance, MAGE or MODD between serum CN1 tertiles (*p* > 0.05), as assessed by the attached continuous glucose monitor for 5–7 days. In concordance, there was also no significant association in fingerprick glucose values between serum CN1 tertiles. 

## 4. Discussion

Since glucose metabolism has been shown to be altered by serum CN1 concentrations in obese subjects, we now assessed whether insulin sensitivity and glucose variability are associated with serum CN1 in type-1 diabetes cohort with CGM. No significant associations were detected. 

Carnosine supplementation has been shown to increase serum insulin concentration in rodent models of obesity and type-2 diabetes [4,13] but also in human [15,17] studies in obese or type-2 diabetic individuals. In db/db mice, insulin resistance was not influenced by carnosine supplementation [4]. Thus, we speculate that carnosine may increase insulin secretion rather than insulin sensitivity. This may explain the lack of association between serum CN1 and insulin in our cohort of individuals with long-standing T1D (mean duration 26 years) with severely impaired/absent endogenous insulin secretion.

Although the association between CN1 and DKD has been shown in numerous studies with patients with type 2 diabetes (T2D), its association with DKD was less clear in type 1 diabetes (T1D). Whereas certain genetic variants of CN1 have been found to be associated with protection against development of DKD in patients with T2D [19,20,21,22,23], a protective effect of the same genetic variants was not found in a study with 1269 patients with T1D [24]. Nevertheless, the possibility remained that CN1 may affect the susceptibility for developing DKD in T1D, as carnosine is known to protect against formation of AGE, ALE and reactive oxygen species that play a role in the pathogenesis of DKD. Furthermore, another study identified genetic variants of CN1 being associated with mortality and progression to end-stage renal disease in T1D [9]. Also, in rodent models of T1D and DKD, supplementation of its substrate carnosine has been shown to prevent podocyte loss and restrain glomerular apoptosis [5,25]. Similarly, carnosine supplementation has been shown to improve glycemic control and renal function in pediatric patients with type 1 diabetes and nephropathy [16]. In this study, we did not observe any association of CN1 concentration with either plasma creatinine, estimated GFR or urinary ACR. However, this may be due to the absence of signs of albuminuria or diabetic kidney disease in the study cohort from which patients with renal impairment were excluded.

To understand the association between CN1 concentration and glucose metabolism, several studies have examined the effect of its substrate carnosine in cell culture. As for insulin secretion, carnosine has been shown to protect pancreatic beta cells from hydrogen peroxide induced cytotoxicity and reduce intracellular reactive oxygen and nitrogen species [26,27], thereby preserving beta-cell function. Additionally, carnosine can scavenge glucolipotoxic radicals in a medium containing glucose, oleic acid and palmitic acid [28], mitigating the effect of glucolipotoxic stress. Beta cells supplemented with carnosine were protected from oxidative and glucolipotoxic stress, resulting in higher insulin secretion compared to cells without carnosine supplementation. Additionally, carnosine intake ifself can stimulate insulin and glucagon secretion within 60–90 min, which is probably mediated by its histidine content [29]. This may facilitate faster normalization of postprandial blood glucose levels and contribute to a reduction in HbA1c. Beyond beta cells, carnosine also protects myotube cells from glucolipotoxic stress, enabling these cells to maintain a normal glucose uptake upon insulin stimulation [28]. Altogether, these findings suggest that carnosine may preserve beta cell function and support normal glucose uptake, as shown in cell culture studies. It should be noted that in the current study population with a mean diabetes duration of 26 years there probably is no residual c-peptide production, i.e., no endogenous insulin secretion, as there is complete destruction of beta cells. Thus, there is no beta-cell function to protect anymore by carnosine. It would be interesting to repeat the study in a population of persons with type 2 diabetes or in persons with T1D but a short disease duration (e.g., <6 months) as there is often still some residual c-peptide production and beta cell function in the first period of the disease.

In our study, we measured blood CN1 concentration, but not the carnosine content in blood or tissue of the patients. Although we could not detect a significant association between endogenous CN1 concentration and glucose metabolism in this type 1 diabetes cohort, we cannot rule out the possibility that supplementation with exogeneous carnosine could influence the glucose metabolism. The cytoprotective effects of carnosine on myotube cells may occur independently of insulin deficiency. Larger randomized controlled studies with sufficient duration and adequate endpoints in both type 1 and type 2 diabetes cohorts are needed to evaluate whether carnosine supplementation could be implemented into clinical practice and ultimately help patients with diabetes. 

It is noteworthy that reduced muscle carnosine content has been observed in patients with type 2 diabetes, but not in those with T1D [30]. This could reduce the protective effect of carnosine supplementation on myotube cells if they are already saturated with carnosine. Even though the reason for the decreased muscle carnosine content only in type 2 diabetes remains unclear, this may explain the differences observed in genetic studies regarding polymorphisms in the CNDP1 gene, which encodes CN1. While a genetic polymorphism associated with a lower carnosinase level has been linked to protection against DKD in type 2 diabetes, this association is less clear in type 1 diabetes. Moreover, beside the leucine repeat polymorphisms, which influence the secretion of the enzyme from cells, certain single nucleotide polymorphisms (SNPs) have been associated with renal outcomes [22,31,32]. However, the impact of these genetic variations on the enzyme function is not well understood. Since we only measured the CN1 enzyme concentration, we do not know about the genetic, translational, and post-translational factors that may influence its activity. 

The results of our study are limited by its observational cross-sectional nature, limited variance of renal function parameters by design, which did not allow assessment of CN1 with respect to renal function, and the strongly controlled patient, which may hamper the translation of these results to a wider population. Furthermore, the accuracy of the blinded sensor used in this study, which measures glucose in interstitial fluid and is expressed as the mean absolute relative difference (MARD) compared to a blood glucose reference method, is slightly lower (9.9%) than that of sensors commonly used in clinical care or research today, where the MARD is typically below 9.0%. While this could theoretically lead to minor discrepancies between sensor-measured levels and blood glucose levels, we believe it would not meaningfully impact our results. The advantages of this study are the homogeneity of the study population, which is controlled for many possible confounders for glucose variability, the precise recording of glucose variability by continuous glucose monitoring for 5–7 days, and a well-suited power with a cohort size of 172 patients. The results of this study may be extrapolated to other Caucasian populations in developed countries. 

In summary, we measured serum CN1 concentration in a well-controlled T1D cohort wearing a blinded CGM sensor for 5–7 days and could not find an association of serum CN1 with glucose variability. 

## Figures and Tables

**Table 1:** Clinical parameters and serum CN1 concentration tertiles.

				CN1 Concentration Tertiles ^b,c^			
	*n*	Descriptives ^a^		Tertile 1 (*n* = 57)	Tertile 2 (*n* = 58)	Tertile 3 (*n* = 57)	*p*-Value
age [years]	172	50.3	(12.5)	49.8 (13.5)	52.3 (12.3)	48.7 (11.7)	0.29
gender [% male]	172	63.0	% female	63.2%	62.1%	61.4%	0.98
BMI [kg/m^2^]	172	26.4	(4.5)	26.2 (4.5)	26.2 (4.2)	26.7 (4.9)	0.80
diabetes duration [years]	172	26.3	(12.3)	25.9 (12.5)	26.7 (12.9)	26.4 (11.6)	0.94
HbA1c [mmol/mol]	172	63.7	(10.2)	65.6 (9.9)	63.3 (10.5)	62.2 (10.1)	0.20
SBP [mmHg]	172	136.7	(18.1)	136.1 (19.5)	137.3 (18.1)	137.5 (16.7)	0.90
DBP [mmHg]	172	79.6	(10.7)	77.8 (11.5)	79.9 (11.7)	81.3 (8.4)	0.22
plasma creatinine [µmol/L]	172	69.3	(12.7)	69. 8 (14.1)	70.6 (13.1)	67.5 (10.6)	0.40
eGFR CKD-Epi [mL/min/1.73 m^2^]	172	96.3	(16.0)	96.1 (16.8)	93.3 (17.4)	99.5 (13.2)	0.12
urinary ACR [mg/mmol]	169	0.9	[1.2]	0.8 [1.3]	0.6 [1.3]	1.0 [1.5]	0.52
Insulin dose [IU/kg body weight]	171	0.7	(0.2)	0.6 (0.2)	0.6 (0.2)	0.7 (0.3)	0.09
continuous glucose monitor							
mean glucose [mmol/L]	158	9.6	(1.9)	10.1 (1.9)	9.6 (1.9)	9.3 (1.9)	0.09
SD of individual blood glucose [mmol/L]	158	3.9	(0.9)	4.0 (0.9)	3.8 (0.9)	3.8 (0.8)	0.32
coefficient of variance [%]	158	40.8	(8.7)	40.4 (8.4)	40.8 (9.6)	41.4 (8.3)	0.84
MAGE [mmol/L]	158	7.8	(2.5)	8.1 (2.3)	7.6 (2.5)	7.7 (2.6)	0.49
MODD [mmol/L]	157	4.1	(1.3)	4.3 (1.3)	4.1 (1.7)	3.9 (1.0)	0.29

^a^ descriptives as mean (SD), median [IQR], or frequency (%); ^b^ tertiles as mean (SD), median [IQR], or frequency (%); ^c^ CN1 concentration tertiles: 0.00 [0.02], 0.12 [0.05], 0.39 [0.27] ng/mL. Abbreviations: body mass index (BMI), glycated hemoglobin (HbA1c), systolic/diastolic blood pressure (SBP/DBP), estimated glomerular filtration rate using the CKD-EPI formula (eGFR CKD-Epi), urinary albumin–creatinine ratio (urinary ACR), international unit (IU), standard deviation (SD), mean amplitude of glucose excursions (MAGE), mean of daily difference (MODD).

## Data Availability

Original data for this study are available upon request from the corresponding authors.
